# Responsiveness to ripasudil may be a potential outcome marker for selective laser trabeculoplasty in patients with primary open-angle glaucoma

**DOI:** 10.1038/s41598-021-85271-w

**Published:** 2021-03-12

**Authors:** Taro Baba, Kazuyuki Hirooka, Hiroki Nii, Yoshiaki Kiuchi

**Affiliations:** 1 Department of Ophthalmology, JA Hiroshima General Hospital, Hiroshima, Japan; 2 Department of Ophthalmology and Visual Science, Hiroshima University, 1-2-3 Kasumi, Minami-Ku, Hiroshima, 734-8551 Japan

**Keywords:** Diseases, Medical research

## Abstract

We examined responsiveness to ripasudil as a potential factor for predicting the effect of selective laser trabeculoplasty (SLT) when performed for primary open-angle glaucoma (POAG). A total of 70 eyes with no history of glaucoma surgery underwent SLT between January 2015 and June 2019. Patients were divided into two groups, with an intraocular pressure (IOP) decrease of 15% or more due to ripasudil administration before SLT defined as the effective group, while an IOP decrease of less than 15% was defined as the non-effective group. Kaplan–Meier survival analysis was performed. A Cox proportional hazards model assessed the influence of baseline factors on the success. Of the 70 eyes evaluated, treatments were effective in 22 and non-effective in 48. Postoperatively, both groups exhibited IOP reductions for up to 24 months. Success ratios at 12 and 24 months after SLT were 43.5% and 18.5% in the effective versus 24.9% and 9.3% in the non-effective group, which were significantly higher in the effective group (*P* = 0.03). Presence of a ripasudil effective eye (*P* = 0.03) was associated with treatment success. Responsiveness to ripasudil may be useful in predicting the therapeutic effect of SLT.

## Introduction

Glaucoma has been demonstrated to be the leading worldwide cause of irreversible vision loss^1^. The primary proven treatment for managing glaucoma is the lowering of the intraocular pressure (IOP). In Japan, the Rho kinase inhibitor, ripasudil (K-115), was approved for use in December 2014. The mechanism of action involves changes in the trabecular meshwork and Schlemm’s canal endothelium, which results in an increase in the aqueous outflow from the main pathway, thereby lowering the IOP^2^.

In 1995, Latina et al. demonstrated the applicability of using selective laser trabeculoplasty (SLT) in the treatment of glaucoma^3^. In addition, it was also reported that irradiation using a Q-switched Nd:YAG laser for a very short time was able to selectively act on the pigmented trabecular meshwork cells, thereby lowering the IOP^2^. The mechanism of action involves increasing the aqueous humor permeability of Schlemm’s canal endothelial cells, which leads to a lowering of the IOP^3^. Over the past two decades, glaucoma management has increasingly begun to use SLT, both as a first-line treatment in newly diagnosed patients and as adjunctive treatment in patients that have not been able to achieve desired IOP levels with topical medications and thus, require additional IOP lowering^4^. Although SLT outcomes have been reported to be relatively favorable^5–7^, far worse outcomes have been reported in other studies^8,9^. In addition, various factors may be able to greatly influence the efficacy of SLT. Therefore, knowledge of the clinical outcome markers of success after the SLT procedure is important in these patients. The aim of the present study was to examine primary open-angle glaucoma (POAG) patients and evaluate the potential of using the responsiveness to ripasudil as an SLT outcome marker.

## Materials and methods

### Patients and examinations

This study retrospectively reviewed the charts of glaucoma patients who underwent SLT at Hiroshima General Hospital between January 2015 and June 2020. The Hiroshima General Hospital Institutional Review Board approved this study protocol, and in accordance with the principles of the Declaration of Helsinki, all patients provided written informed consent as well as the standard consent for SLT.

Patients enrolled in the study had a history of IOP greater than 22 mmHg, an open-angle, and glaucomatous visual field defects. To be included in the study, patients were required to be older than 20 years of age at the time of their first recorded SLT and to have had at least 3 months of post-SLT follow-up. Patients underwent SLT treatment when their IOPs were above maximal goal pressures. In addition, all patients included in this study were under maximally tolerated medical therapy. Exclusion criteria included having any significant ocular diseases, history of previous glaucoma surgery, history of SLT, or eyes having undergone intraocular surgery for up to 6 months prior to the SLT. IOP was measured by Goldmann applanation tonometry.

### Laser procedure

For all of the procedures performed at our institute, patients underwent SLT with the Q-switched frequency-doubled 532 nM Nd:YAG laser (Laserex Tango; Ellex Medical Lasers, Adelaide, Australia). All patients were treated with apraclonidine (Novartis Pharmaceuticals UK Limited, London, United Kingdom) immediately before and after the SLT procedures. After administering a drop of 4% lidocaine, we used a Latina single mirror lens (Ocular Instruments, Bellevue, WA) to visualize the angle. Nonoverlapping laser spots were applied to 360° of the angle. Laser spot size was 400 μm with a pulse width of 3 ns. Irradiation was performed using the minimum energy required to generate bubbles. None of the patients were administered any postoperative steroid eye drops. All patients were instructed to continue the same IOP-lowering medications.

### Main outcome measures

All patients enrolled in the study received ripasudil prior to undergoing SLT. Ripasudil was administered before SLT for at least two months (effective group; 14.0 ± 12.8 months, range 2–47 months, non-effective group; 9.9 ± 7.6 months, range 2–34 months). IOP was evaluated at least twice after the ripasudil administration, with the average IOP compared before (twice) and after (twice) the ripasudil administration. Following the administration, ripasudil was defined as being effective when there was greater than 15% reduction in the IOP. Patients were then divided into the effective or non-effective groups. If both eyes were treated, patient data from only the first eye operated on were selected and used for the study.

The primary outcomes measured included the change in the IOP and the treatment success and survival. Baseline IOP was defined as the last IOP measurements obtained before the SLT. Kaplan–Meier survival curves and the log-rank test were used to compare the outcomes between the effective and non-effective groups. We defined SLT treatment failure as one or more of the following: (1) need for a subsequent glaucoma procedure, including repeat SLT; (2) an increase from baseline in the number of IOP-lowering medications; or (3) change of the IOP on any two consecutive visits: IOP > 21 mmHg or IOP reduction < 20% from baseline. Administration of repeat SLT or additive IOP-lowering medication was based on the clinician’s (H.N.) decision. Influence of baseline factors on the success were assessed using Cox proportional hazards analysis, univariate analysis, and multivariate analysis. The following factors were tested for associations with failure: age, total power, preoperative IOP, preoperative number of IOP-lowering medications, and effectiveness of ripasudil. A Goldmann applanation tonometer was used to examine the IOP. The IOP on the day of SLT was defined as the preoperative IOP.

### Statistical analysis

Student’s *t* test for the continuous variable and a chi-square test for categorical variables were used to compare the clinical characteristics between the effective and non-effective groups. The Kaplan–Meier survival curve and the log-rank test were used to compare the outcomes between the effective and non-effective groups. Cox proportional hazards regression model analysis was used to examine the predictive value of the significant factors. The following factors were tested for associations with SLT failure: age, total laser power, preoperative IOP, preoperative number of IOP-lowering medications and effectiveness of ripasudil. Multivariate factors were selected from variants with a probability value of less than 0.05, as shown by the univariate analysis. All data are reported as the mean ± standard deviation (SD). All statistical analyses were conducted using JMP software version 11 (SAS Inc., Cary, NC). P values less than 0.05 were considered statistically significant.

### Ethics approval

This study was approved by the Institutional Review Board of Hiroshima General Hospital.

## Results

Table 1 presents the patient baseline characteristics. A total of 70 patients (70 eyes) were analyzed in the study, with 22 eyes in the effective group and 48 eyes in the non-effective group. Mean age was 72.1 ± 11.6 years and 70.1 ± 8.3 years in the effective and non-effective groups, respectively (*P* = 0.30). There were no significant differences noted for gender (*P* = 0.21), lens status (*P* = 0.06), total laser power (*P* = 0.26), or preoperative IOP (*P* = 0.78). However, there were significant differences noted for the IOP before administration of ripasudil (*P* = 0.01) and the number of IOP-lowering medications (*P* = 0.02). The mean follow-up period was 10.4 ± 13.0 months (range 3–56 months) in all cases, 14.0 ± 10.4 months (range 3–56 months) in the effective group and 7.1 ± 9.8 months (range 3–44 months) in the non-effective group. The duration from the beginning of the ripasudil administration to the SLT procedure was 16.7 ± 13.2 months in the effective group and 11.8 ± 8.9 months in the non-effective group. Seven patients in the effective group and 19 patients in the non-effective group ceased ripasudil administration before starting SLT due to side effects. However, there have been no patients lost during the follow-up.

**Table 1:** Clinical characteristics.

	Effective group (n = 22)	Non-effective group (n = 48)	*P* value
Age (years)	72.1 ± 11.6	70.1 ± 8.3	0.30
Gender (M/F)	15/7	25/23	0.21
Lens status (phakia/pseudophakia)	17/5	25/23	0.06
Total laser power (mJ)	56.2 ± 16.0	61.2 ± 18.3	0.26
Duration of glaucoma (mo)	105.4 ± 50.5	97.3 ± 51.7	0.65
Duration of ripasudil treatment before SLT (mo)	14.0 ± 12.8	9.9 ± 7.6	0.33
Duration from the beginning of ripasudil treatment to SLT (mo)	16.7 ± 13.2	11.8 ± 8.9	0.15
Preoperative IOP (mmHg)	19.9 ± 5.9	21.1 ± 7.1	0.78
IOP before administration of ripasudil (mmHg)	19.2 ± 4.5	16.8 ± 5.6	0.01
Preoperative IOP-lowering medications	4.4 ± 1.1	3.8 ± 1.1	0.02

*M* male, *F* female, *SLT* selective laser trabeculoplasty, *IOP* intraocular pressure.

**Figure 1:**
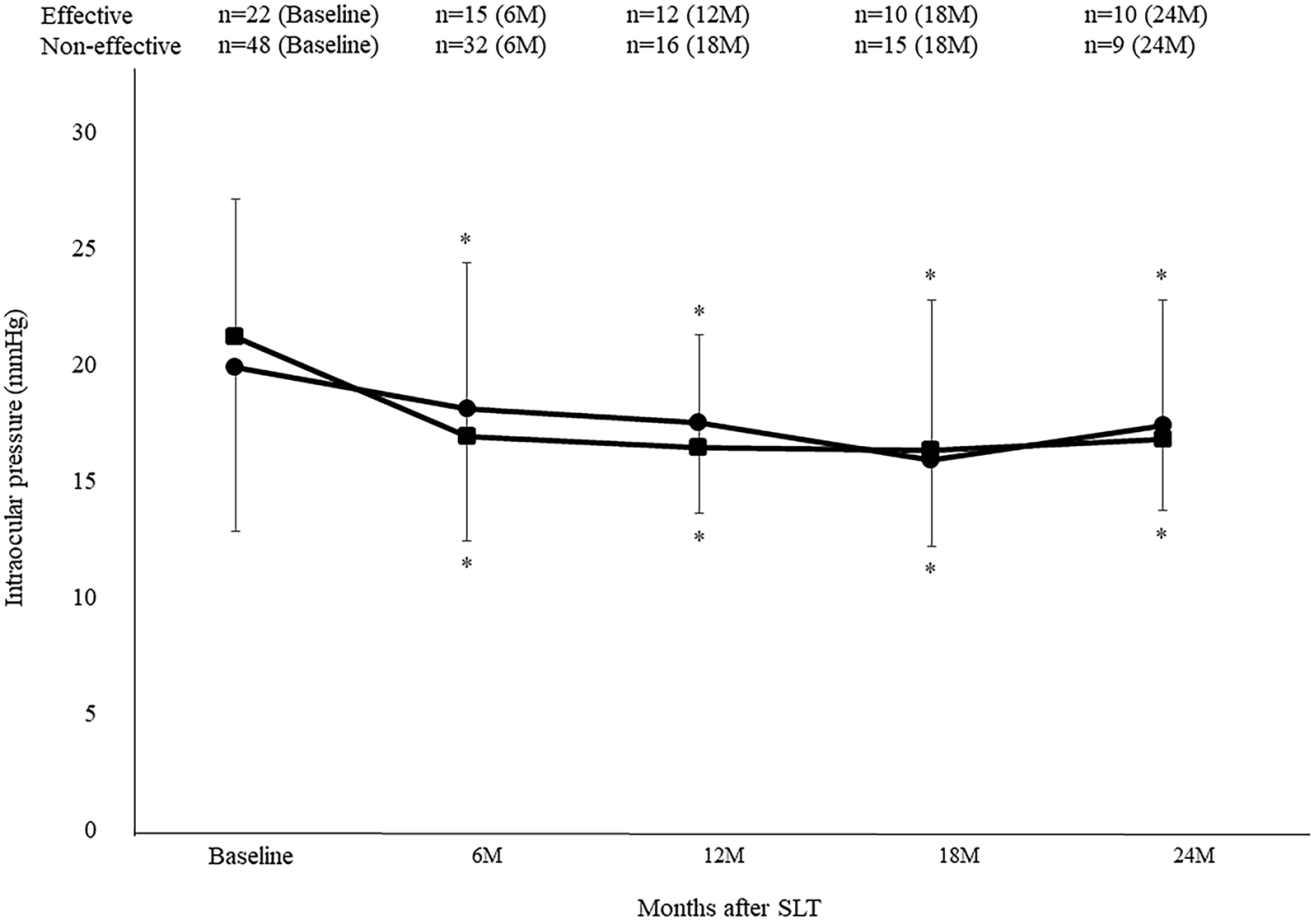
Mean intraocular pressure following selective laser trabeculoplasty in the effective and non-effective groups. Intraocular pressure was significantly reduced in both groups as compared with baseline. *P < 0.05 as compared with baseline. ●: effective group, ■: non-effective group.

After the SLT, both groups exhibited a decrease in the IOP (Fig. 1). The mean IOP in the effective group was 19.9 ± 5.9 mmHg (n = 22) at baseline, while it was 18.1 ± 7.5 (n = 15), 17.5 ± 4.9 (n = 12), 15.9 ± 6.5 (n = 10), and 17.4 ± 6.0 (n = 10), at 6, 12, 18 and 24 months, respectively. In the non-effective group, the IOP was 21.1 ± 7.1 mmHg (n = 48) at baseline, while it was 16.9 ± 5.7 (n = 32), 16.4 ± 3.9 (n = 16), 16.3 ± 3.7 (n = 15), and 16.8 ± 3.7 (n = 9), at 6, 12, 18 and 24 months, respectively.

**Figure 2:**
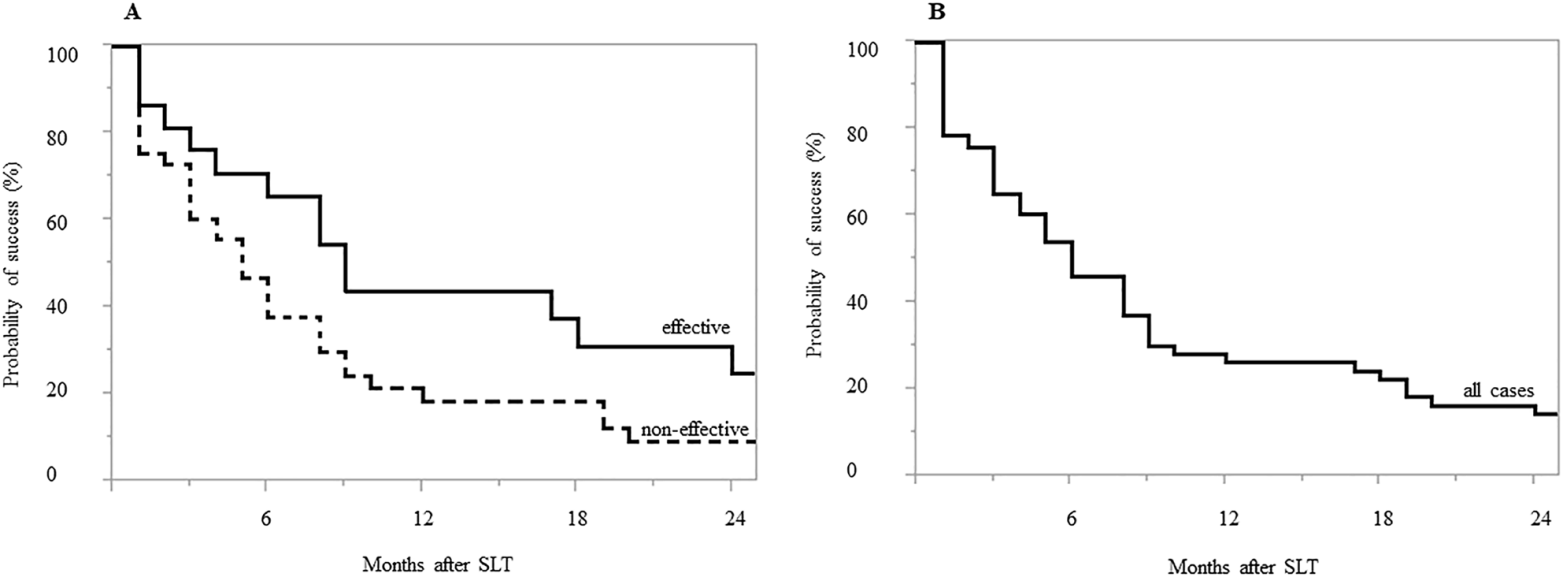
Kaplan–Meier survival curve of surgical outcomes in the ripasudil effective vs. non-effective eyes (**A**) and all cases (**B**). Significant differences were noted for the cumulative probability of success between the effective and non-effective groups (*P* = 0.03).

**Figure 3:**
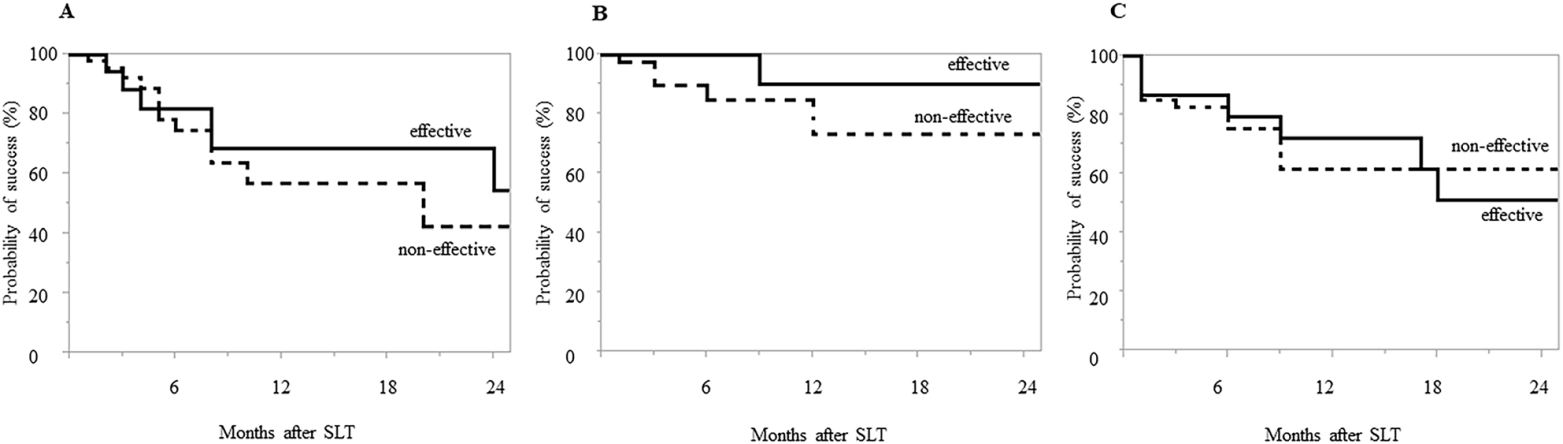
Kaplan–Meier survival curve of surgical outcomes in the ripasudil effective vs. non-effective eyes. (**A**) Need for a subsequent glaucoma procedure, including repeat SLT. (**B**) An increase from baseline in the number of IOP-lowering medications. (**C**) Change of the IOP on any two consecutive visits: IOP > 21 mmHg or IOP reduction < 20% from baseline. There were no significant differences noted between the groups for any of the criteria.

Figure 2 shows the Kaplan–Meier survival-curve analysis. Surgical success rates were 65.3% and 37.8% at 6 months, 43.5% and 18.5% at 12 months, 31.3% and 18.5% at 18 months, and 24.9% and 9.3% at 24 months in the effective and non-effective groups, respectively (*P* = 0.03) (Fig. 2A). The survival rates at 6, 12, 18 and 24 months were 46.0%, 26.4%, 22.3% and 14.2% in all cases, respectively (Fig. 2B). We additionally evaluated the survival rate for each of the following criteria; (1) need for a subsequent glaucoma procedure, including repeat SLT; (2) an increase from baseline in the number of IOP-lowering medications; or (3) change of the IOP on any two consecutive visits: IOP > 21 mmHg or IOP reduction < 20% from baseline. Surgical success rates were 68.3% and 56.8%, 90.0% and 72.9%, and 71.8% and 61.4% for criteria (1), (2), and (3) at 12 months and 54.6% and 42.6%, 90.0% and 72.9%, and 51.3% and 61.4% for criteria (1), (2), and (3) at 24 months in the effective and non-effective groups, respectively (criteria (1): *P* = 0.64, criteria (2): *P* = 0.09, criteria (3): *P* = 0.97) (Fig. 3). The observed reasons for failure that were noted on the date of failure included; an increase in the number of IOP-lowering medications (effective group; n = 1, 4.5%, non-effective group; n = 7, 14.6%), IOP > 21 mmHg or IOP reduction < 20% (effective group; n = 7, 31.8%, non-effective group; n = 12, 25.0%), and the requirement for a further glaucoma procedure (effective group; n = 7, 31.8%, non-effective group; n = 20, 41.7%).

Table 2 presents the univariable associations with treatment failure. Non-effective ripasudil results were significantly associated with treatment failure.

**Table 2:** Univariable associations with failure of selective laser trabeculoplasty.

Factors	Univariate		
	RR	HR (95% CI)	*P* value
Age per year	0.98	0.952–1.002	0.08
Total power per mJ	1.00	0.985–1.020	0.72
Preoperative IOP per mmHg	1.01	0.968–1.059	0.53
No. Preoperative IOP-lowering medications	0.85	0.693–1.061	0.15
IOP before administration of ripasudil	1.00	0.950–1.050	0.94
Duration of glaucoma per month	1.00	0.996–1.007	0.57
Effectiveness of ripasudil	0.53	0.285–0.965	0.04

*CI* confidence interval, *RR* risk ratio, *HR* hazard ratio, *IOP* intraocular pressure.

## Discussion

The present study evaluated the success rate of SLT in ripasudil effective and non-effective eyes. In the ripasudil effective eyes, there was a significantly higher cumulative probability of SLT success after the procedure as compared to that observed in the non-effective eyes. To the best of our knowledge, this is the first report to provide details on the relationship between ripasudil effective eyes and SLT success.

Potential predictive factors of success following SLT have been examined in a number of previous studies. Since there has been a growing interest in using SLT as a first-line therapy, the elucidation of prediction factors for SLT success has become an increasingly important topic^10^. A significant association has been found between the preoperative IOP and SLT success in several previous studies. While most of these studies determined that a higher IOP was associated with treatment success^6,8,11–14^, one other previous study reported that a higher IOP was associated with failure^7^. In the present study, we additionally found that there was no association between the preoperative IOP and SLT success. While a high preoperative IOP could potentially be advantageous with regard to the absolute magnitude of the IOP reduction, there is a chance it could additionally be disadvantageous if this indicates that larger reductions in the IOP are required in order to achieve the target IOP.

The most remarkable prediction factor for SLT success in the present study was the presence of ripasudil effective eyes. Several other studies have also reported that induced changes of the trabecular meshwork cellular activities were associated with the IOP-lowering effect of the Rho kinase inhibitor^15,16^. SLT lowers the IOP by inducing biological changes in the trabecular meshwork, which subsequently leads to an increased aqueous outflow^17^. We recently reported that ripasudil could potentially be used as a trabeculotomy outcome marker^18^. Relief of outflow resistance in the trabecular meshwork is the primary target of trabeculotomies that are performed in order to reduce the IOP. The effectiveness of the SLT in the ripasudil effective eyes could be due to the consistency between the laser target and the modulating lesion.

Although there is considerable variation in the reported IOP-lowering efficacy of SLT, the results of the present study are in line with data reported for the majority of eyes that have demonstrated an initial response with a gradual decline in efficacy over time^19^. The 29.1% treatment success found for our patients at 12 months is in agreement with other previous studies that included medically treated glaucoma, which generally found there was a 21.7–46% success at 12 months^7,14,20,21^.

Previous studies of Japanese patients who were already on maximum medical therapy reported that the IOP decreased from 2.6 to 3.1 mmHg or approximately 15–16% from baseline after the administration of ripasudil^22–25^. Results of the phase 2 study of ripasudil demonstrated that the mean IOP reduction from baseline was approximately 10% in the placebo group^26^. Therefore, we defined a greater than 15% reduction in the IOP after ripasudil administration as indicating effectiveness.

Although the preoperative IOP was similar in both groups, the IOP before the administration of ripasudil in the effective group was significantly higher than that found in the non-effective group. Other previous studies reported that the IOP reduction after the administration of ripasudil was associated with higher baseline IOP levels^27,28^. Our results support the findings of these previous studies.

Although the average IOP after SLT was similar between the effective and non-effective groups, the success rate for the effective group was significantly higher than that of non-effective group. After the administration of additional IOP-lowering medications, repeat SLT, or additional glaucoma surgery, we excluded patients who received additional treatments before calculating the mean IOP. After these exclusions, we determined that the mean IOP was continuously lower, especially in the non-effective group.

There were some limitations for the present study. First, patient selection may have been biased due to the retrospective nature of this study. Since this was a retrospective clinical study, with the exception for ripasudil, it was hard to confirm the response of other IOP-lowering medications and the IOP in subjects without any medical treatment based just on our clinical records. However, if IOP-lowering medications such as the prostaglandin analog or a β-blocker did not decrease the IOP to that expected, we usually did not continue these administrations. Therefore, we assumed that all of included patients were IOP responders to other medications. Additional prospective and randomized studies will need to be conducted in order to support the present results. Second, the degree of the angle pigmentation was not recorded in this study and therefore, the potential role of this variable could not be explored. Third, we applied SLT to the 360° circumference of the drainage angle. There are multiple previous studies that reported finding that a greater angle treatment resulted in a greater response^20,29^. Therefore, it is not clear whether the predictor of success identified in the present study, the ripasudil effective eyes, would additionally be relevant to SLT when applied to a 180° circumference or other various ranges. However, we recently reported that during a trabeculotomy, a 120° opening of the trabecular meshwork was effective for achieving an IOP < 21 mmHg in ripasudil effective POAG eyes^18^. Thus, the ripasudil effective eye may be a predictor of SLT success when applied to a 180° circumference or other various ranges. Fourth, as we only examined a small number of subjects in the present study, a further study with a larger number of subjects will need to be undertaken to address this issue.

In conclusion, we have demonstrated that SLT is an effective method for lowering the IOP. The presence of a ripasudil effective eye appears to significantly predict the success of the procedure.

## Data Availability

All data generated or analyzed during this study are included in this published article.
